# Evaluating the Emergency Surgery Score (ESS) in Predicting Postoperative Outcomes Following Emergency Laparotomy: Insights From an Indian Tertiary Center

**DOI:** 10.7759/cureus.56455

**Published:** 2024-03-19

**Authors:** Srishti Bhowmik, Chandra B Singh, Sushanto Neogi, Sarmista Roy

**Affiliations:** 1 Department of Surgery, Maulana Azad Medical College, New Delhi, IND

**Keywords:** emergency laparotomy, indian study on emergency surgery, predicting postoperative course, surgical acute abdomen, emergency surgery score, postoperative course

## Abstract

Aims and objectives: To determine the predictive value of Emergency Surgery Score (ESS) with regard to mortality and morbidity rates of patients undergoing emergency laparotomy.

Method: The ESS ranging from 0 to 29 is an extensive risk calculator based on 22 variables including important parameters like demographics, preoperative treatment, comorbidities, and laboratory values. Twenty patients who underwent emergency laparotomy were preoperatively assessed and ESS was calculated for each. After establishment of diagnosis and resuscitation, the patient was taken up for emergency laparotomy. Postoperatively, patients were monitored clinically as well as with laboratory and radiological investigations as per case needed till discharge and further followed up physically in OPD/ward or interviewed telephonically for 30 days on a weekly basis. Incidence of mortality and morbidity in terms of postoperative complications, ICU admission, reoperation and readmission among the cases occurring within 30 days of procedure were recorded.

Results: ESS correlated well with the outcome in the current study, 10 out of 14 patients with score less than 8 were discharged without any complications. Mean ESS was higher among non-survivors. Ability of ESS to predict postoperative mortality, morbidity and ICU stay was proven statistically with c-statistics of 0.853, 0.84, 0.879 respectively. ESS was found to be a good predictor for the development of postoperative lower respiratory tract infection (LRTI) (c-statistic=0.828), sepsis (c-statistic=0.867), disseminated intravascular coagulation (DIC) (c-statistic=0.805), acute kidney injury (AKI) (c-statistic=0.804). ESS showed poor correlation with reoperation and readmission rates.

Conclusion: The current study underscores the critical importance of employing risk stratification through ESS for patients undergoing emergency laparotomy. By employing ESS, healthcare professionals can accurately anticipate resuscitation requirements and stabilize patients preoperatively. This proactive approach enables the identification and optimization of patients unsuitable for immediate surgery, facilitating informed decisions on targeted treatment, surgical intervention, and postoperative care pathways.

## Introduction

Patients requiring emergency general surgery (EGS) procedures represent a discrete, high-risk population with frequent poor outcomes. Out of all emergency admissions to surgical units, 7.1% of patients require EGS, chiefly laparotomy [1]. In low- and middle-income countries (LMICs), at least 60% of the surgical operations performed are for emergencies [2].

Multiple recent studies have shown that emergency surgeries are associated with considerably higher morbidity and mortality compared to elective surgeries, even when they are operated on after physiological optimization of their altered hemodynamic and maximum stabilization. Patients undergoing EGS are approximately 2.5 times more likely to experience a significant complication and have a six-fold increase in mortality as compared to non-EGS patients. EGS cases comprise 11% of all general surgery operations, yet account for 47% of mortalities and 28% of complications [3].

The Emergency Surgery Score (ESS) has been recently devised by Sangji et al. as the EGS equivalent of the Trauma Injury Severity Score due to an increasing need for a risk assessment tool specific to emergency laparotomy [4]. Currently, ESS is the only existing risk estimation tool for emergency surgical patients that has been shown to predict accurately the probability of postoperative mortality, morbidity, and complications including infections [5-7].

Using ESS, patients unsuitable for operation can be identified and optimized beforehand; well-informed decisions can be taken regarding treatment, operation, and postoperative care. Targeted care can minimize the risk of postoperative complications - avoiding mortality and morbidity and saving money.

Realizing the utility of an emergency scoring system in mortality prediction in surgical patients, the current study was undertaken to evaluate the surgical outcome in the form of mortality and postoperative morbidities like duration of hospital stay, ICU stay, wound infection, wound dehiscence, chest infection anastomotic leak, requirements of revision surgery and thereby finding its general and widespread applicability in our hospital setup. This will help us in propagating its use across the institutions and help us in assessing the state of health with which our patients present to emergency surgical units.

## Materials and methods

A prospective observational study was conducted in the Department of Surgery, Maulana Azad Medical College, and associated Lok Nayak Hospital, New Delhi over a period of 12 months. The study was approved by the Institutional Ethics Committee and written informed consent was taken from all the patients.

A total of 32 patients who presented to Lok Nayak Hospital and underwent EGS for acute abdominal diseases in the surgical emergency department were screened for the study. All patients aged 18 years or above who underwent emergency laparotomy were included in the study. Patients of trauma requiring laparotomy and pregnant ladies were excluded from the study. Out of the 32, four patients were excluded from the study as they did not meet the inclusion criteria. From the remaining 28 patients who met the inclusion criteria of the study, eight were excluded based on exclusion criteria. The ESS ranging from 0 to 29 is an extensive risk calculator based on 22 variables as shown in Table 1.

**Table 1:** Parameters in ESS ESS: Emergency Surgery Score, BMI: Body mass index, COPD: Chronic obstructive pulmonary disease, SGOT: Serum glutamic oxaloacetic transaminase, WBC: White blood cells

Variable	Points
Demographics age >60y	2
White race	1
Transfer from outside emergency department	1
Transfer from an acute care hospital inpatient facility	1
Co-morbidities ascites	1
BMI <20kg/m^2^	1
Disseminated cancer	3
Dyspnoea	1
Functional dependence	1
History of COPD	1
Hypertension	1
Steroid use	1
Ventilator requirement within 48h pre-operatively	3
Weight loss >10% in the preceding 6mo	1
Laboratory values albumin <3.0U/L	1
Alkaline phosphatase >125U/L	1
Blood urea nitrogen >40mg/dL	1
Creatinine >1.2mg/dL	2
International normalized ratio >1.5	1
Platelets <150x10^3^/mcL	1
SGOT >40U/L	1
Sodium >145mg/dL	1
WBC·10^3^/mcL <4.5	1
>15 and ≤25	1
>25	2
Maximum score	29

The primary endpoint of the study was to assess the correlation of ESS in predicting mortality among the cases occurring within 30 days of the procedure. Secondary endpoints were ESS correlation with the incidence of ICU admission, postoperative complications, reoperation, and readmission within 30 days of operation. All patients enrolled in the study underwent routine preoperative workup and optimization for emergency laparotomy. Patient workup included complete history and examination, blood tests (complete blood count, kidney function test with electrolytes, prothrombin time-international normalized ratio (PT-INR), liver function test with alkaline phosphatase (ALP), serum albumin) for patient management and ESS calculation before starting fluid resuscitation. Radiological investigations (chest X-ray, abdominal X-ray, contrast-enhanced computed tomography of the abdomen as and when needed) were done. ESS was calculated preoperatively. After complete workup, establishment of diagnosis, and resuscitation, the patient was taken up for emergency laparotomy. Intraoperative findings were noted. Postoperatively, patients were monitored clinically as well as with laboratory and radiological investigations as required till discharge and further followed up physically in the OPD/ward or interviewed telephonically for 30 days on a weekly basis. A note was made for every case undergoing emergency laparotomy with mortality, postoperative ICU requirement, re-operation, and readmission within 30 days of emergency laparotomy. All the postoperative complications within 30 days of operation were recorded and treated appropriately.

The sample size was calculated using the formula for prevalence study, using postoperative mortality as the primary endpoint. In searching the published literature, mortality was seen in 13% of patients undergoing emergency laparotomy in India according to a study by Gejoe et al. in 2017 [7]. Taking the confidence interval as 95% and the precision of the study as 5%, the total sample size was calculated as 173. However, due to constraints of time and the ongoing pandemic situation, the sample size was taken to be 20.

Data were entered in MS Excel (Microsoft® Corp., Redmond, WA) and analyzed using the Statistical Package for the Social Sciences (IBM SPSS Statistics for Windows, IBM Corp., Version 25.0, Armonk, NY). Demographic, clinical, preoperative, and/or postoperative complications on each patient were entered into a standard Performa. Each patient's postoperative outcome/mortality was compared to determine the significance of illness on postoperative complications and mortality. Categorical variables were presented in number and percentage (%) and continuous variables were presented as mean ± SD and median. A p-value of <0.05 was considered statistically significant.

## Results

​Acute abdomen was common in the age group of 21-30 years and 41-50 years with a mean age of 40.6 years and a lower mean age (37 years) of survivors than of non-survivors (47 years). Perforation peritonitis was the most common cause of acute abdomen among the study population (60%) while Tuberculosis was the most common underlying disease (8/20).

**Figure 1:**
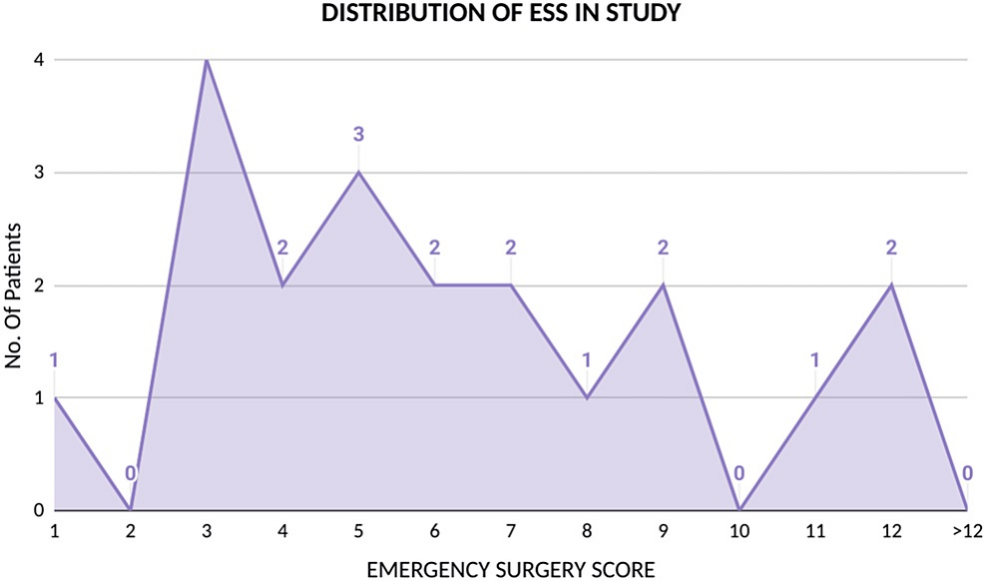
Distribution of ESS in the study ESS: Emergency Surgery Score

​The minimum ESS was 1 and the maximum was 12 with a mean of 6.15. Maximum patients (14 patients out of 20) were in low ESS (0-7) as shown in Figure 1. For survivors, the mean ESS was 5.06 whereas for non-survivors it was 9.4. ESS correlated well with the outcome in the current study; 10 out of 14 patients with scores less than 8 were discharged without any complications.

**Figure 2:**
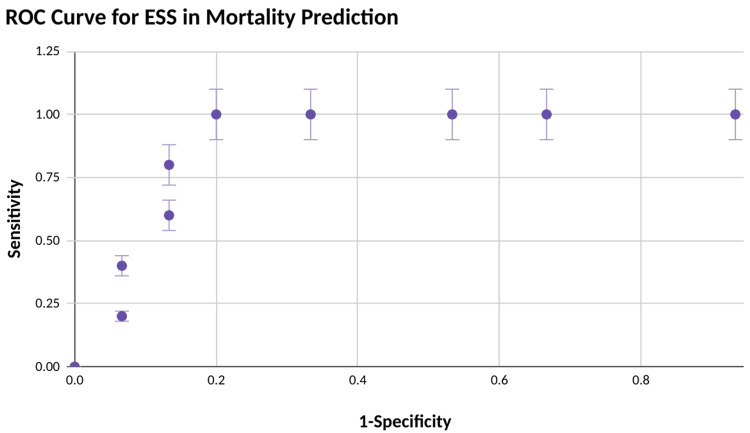
ROC curve for Emergency Surgery Score calculation in mortality prediction ROC: Receiver operator characteristics

As seen in Figure 2, the receiver operator characteristics (ROC) curve of the ESS for predicting mortality shows ESS to be a good predictor for mortality with area under cover (AUC) = 0.853. ​Mortality was equal among females and males. A cut-off value of 7 shows a sensitivity of 80% and a specificity of 87% in predicting mortality for patients undergoing emergency laparotomy with a p-value of 0.013 (statistically significant). Functional dependence, weight loss >10% in the preceding six months, and serum ALP levels >125U/L were found to have a significant effect on postoperative mortality. Lower respiratory tract infection (LRTI), wound dehiscence, sepsis, acute kidney injury (AKI), thrombocytopenia, disseminated intravascular coagulation (DIC), and ICU stay in the postoperative period were found to be significantly associated with mortality.

**Figure 3:**
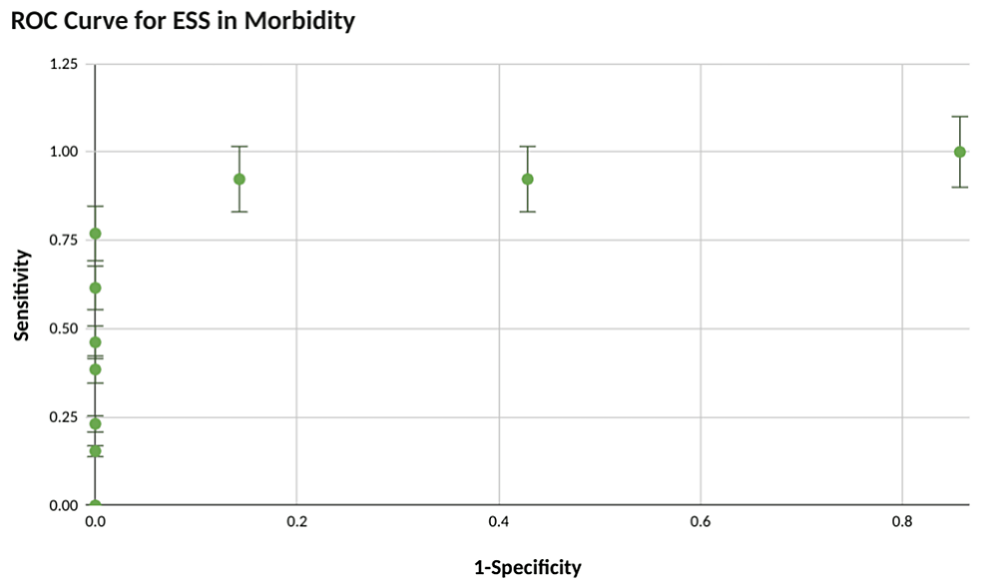
ROC curve for Emergency Surgery Score calculation in morbidity prediction ROC: Receiver operator characteristics

**Figure 4:**
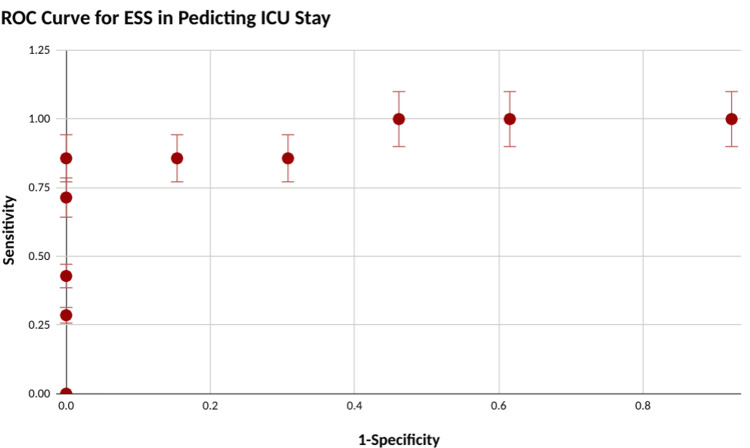
ROC curve analysis for Emergency Surgery Score calculation in the prediction of ICU stay ROC: Receiver operator characteristics, ESS: Emergency Surgery Score

The ability of ESS to predict postoperative morbidity and ICU stay was proven statistically with c-statistics of 0.84 and 0.879 respectively as shown in Figures 3-4. ​Surgical site infection was the most common postoperative complication developed in 50% of patients followed by LRTI in 45%. Wound dehiscence and sepsis developed in 25% of cases each. A cut-off of 7 was found which could accurately predict postoperative morbidity (p-value = 0.04) and ICU stay (p-value = 0.00018) both statistically significant.

ESS was found to be a good predictor for the development of postoperative LRTI (c-statistic=0.828), sepsis (c-statistic=0.867), DIC (c-statistic=0.805), and AKI (c-statistic=0.804). ESS showed a poor correlation between reoperation and readmission rates.

## Discussion

The severity assessment of a disease condition is useful for early-priority treatment, and it reduces morbidity and mortality. High severity scores are usually associated with high morbidity and mortality, therefore these patients may require more intensive treatment than those with low scores.

This was an observational study to categorize the patients undergoing exploratory laparotomy for emergency surgical conditions by calculating their ESS and assessing its predictive value. The surgeon was blinded for the ESS which was calculated for each patient at the admission. The decision regarding the particular operative procedure done was a subjective decision based on the resources available at the operation theater, the general condition of the patient, the condition of the bowel, and purulent contamination. The outcome of the patient was studied and correlated with the ESS calculated at admission to achieve the predictive value of the score regarding the outcome.

In our study, 20 patients who underwent emergency laparotomy were included with ages ranging from 18 to 72 years. The median age of the study population was 40 years. Sex distribution was unequal with a male-to-female ratio of 2:3. The commonest cause for perforation in this study was tubercular perforation (50%) followed by duodenal and gastric (33.33%) followed by large bowel and appendix (16.67%). This contrasts with a study conducted by Agarwal et al. and various other studies by Afridi SP et al. in 2008, Jhobta RS et al. in 2006, Dorairajan et al. In all these studies the most common cause of perforation peritonitis was acid peptic disease with incidences of 39%, 32%, 44.9%, and 21.6% respectively [8,9]. A study from Pakistan had 43% causes due to tuberculosis and typhoid, which highlights the role of infectious pathology as a leading cause of perforation peritonitis in the developing world [10]. This result was quite similar to our study result.

Age as a determinant of outcome

In our study, the mean age of patients was 40.60 years. Maximum cases were in the third and fifth decades (25% each). Maximum mortality was observed in the fifth decade (40%).

John Bohenen studied the effect of age as a risk factor for mortality (in the case of abdominal sepsis) and got the result that patients of less than 50 years of age had 17% mortality whereas those over 50 years had a 45% death rate [11]. In our study less than 40 years old had a mortality of 10%, while more than 40 years had a mortality of 40% which was corroborative with the previous results. Pointing et al. and Frank B. Cerra et al. studied the effects of sepsis and got the result that mean age for non-survivors was higher than survivors [11]. Our studies gave similar results with a lower mean age (37 years) of survivors than of non-survivors (47 years).

ESS and mortality

**Figure 5:**
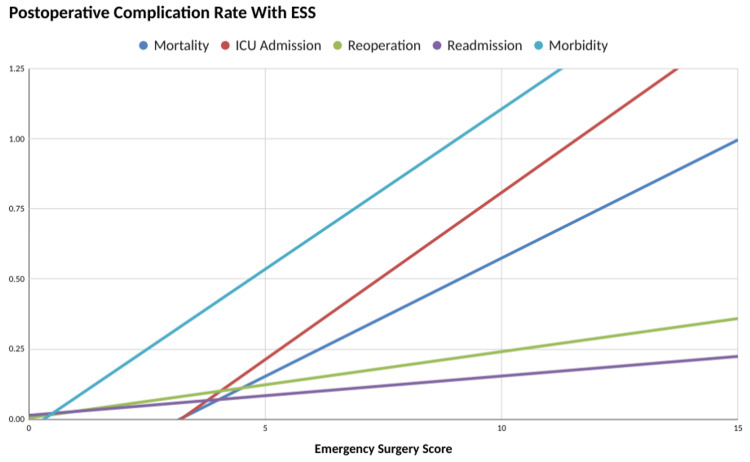
Postoperative complication rate on y-axis with ESS on x-axis ESS: Emergency Surgery Score

We observed a maximum number of cases in our study that had an ESS score at admission of 3-5. In this group, we observed 25% (one out of four patients) mortality. Maximum mortality was observed in ESS score greater than 7 with 66.67% mortality. The mean ESS score was 6.15. For survivors, it was 5.06 whereas for non-survivors it was 9.4. There was no death in patients who scored 0-6. Figure 5 shows the correlation between ESS with postoperative complications.

Sangji et al. derived and validated the ESS scoring system for the first time in 2012. A total of 19,552 cases undergoing emergency laparotomy were studied. Mortality rate increased from 0% to 36% in patients who scored 0 to 11 and finally 100% at 22 with a c-statistic of 0.86 [4]. Peponis et al. also worked on ESS and reported that ESS correlated well with mortality (c-statistic = 0.84); 0.4%, 39%, and 100% mortality at scores of 1, 11, and 22 respectively [12]. Kaafarani et al. recently demonstrated the efficacy of ESS in a prospective multi-centric study in predicting 30-day mortality. The 30-day mortality was 14.8%. ESS gradually and accurately predicted 30-day mortality; 3.5%, 50.0%, and 85.7% of patients with ESS of 3, 12, and 17 died after surgery, respectively, with a c-statistic of 0.84. It concluded ESS can be useful for perioperative patient and family counseling, triaging patients to the ICU, and benchmarking the quality of EGS care [13].

ESS and morbidity

**Figure 6:**
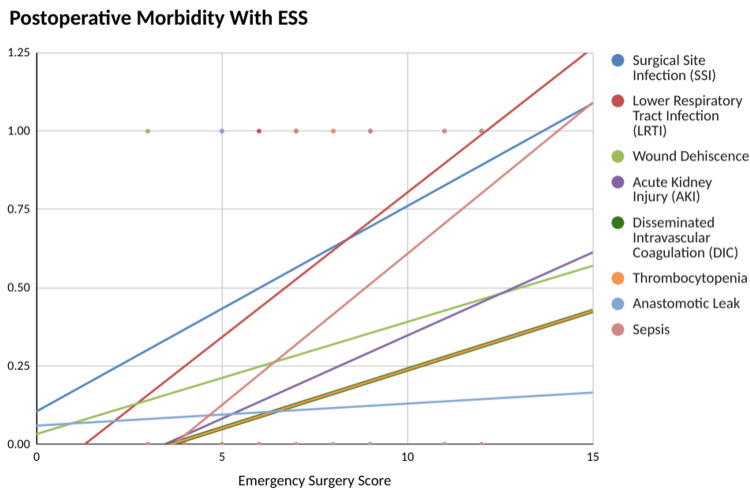
Postoperative morbidity rate on y-axis with ESS on x-axis ESS: Emergency Surgery Score

Postoperative complications as shown in Figure 6 in the form of surgical site infection, LRTI, and more were seen in 65% of the patients enrolled. The majority of postoperative complications in our study was surgical site infection, seen in 50% of the patients followed by LRTI in 45% of the patients. Sepsis and wound dehiscence were seen in 25% of the patients each. Fifteen percent of the patients developed AKI postoperatively while 10% of the patients showed anastomotic leak, DIC, and thrombocytopenia each. With increasing ESS, the postoperative complication rate also increased (c-statistic = 0.820).

Peponis et al. extrapolated ESS to predict 30-day morbidity in patients undergoing emergency laparotomy. This study demonstrated ESS correlated well with morbidity (c-statistic = 0.74); morbidity rates steadily increased from 13% at score 0 to 58% at score 7 and 79% at score 11 after which the complication rates plateaued [12].

Nandan et al. in 2017 also validated the efficacy of ESS in predicting postoperative complications in patients undergoing EGS. It reported out of 37,999 cases enrolled, 14,446 (38%) resulted in at least one complication within a period of 30 days. The observed 30-day complication rates consistently increased from 7% to 53% to 91% at scores of 0, 7, and 15, respectively, after which it plateaued at a mean of 92% for ESS>15, with a c-statistic of 0.78. On multivariable analyses, each of the 22 ESS components independently predicted the occurrence of postoperative complications. Out of the complications reported, the most common was surgical site infection (12.9%) followed by pneumonia (7.3%) [5]. Our study reported a similar trend.

Han et al. reported a total of 90,412 patients out of which 22% developed one or more postoperative infections, the most common of which was sepsis/septic shock (12.2%), followed by surgical site infection (9%), and pneumonia (5.7%). The ESS accurately predicted infectious complications; postoperative infections developed in 7% of patients with ESS=1, in 24% of patients with ESS=5, and in 49% of patients with an ESS=10. The c-statistics for overall postoperative infection was 0.73, postoperative sepsis/septic shock was 0.75, and pneumonia was 0.80. It recommended the use of ESS in estimating the risk of infections in patients undergoing emergency laparotomy within a period of 30 days [6]. Kaafarani et al. also demonstrated the efficacy of ESS in predicting 30-day postoperative morbidity in a study enrolling 1,649 patients. The 30-day complication rate was 53.3%. ESS gradually and accurately predicted complications; 21.0%, 57.1%, and 88.9% of patients with ESS of 1, 6, and 13 developed postoperative complications, with a c-statistic of 0.74 [13].

ESS and ICU admission

In our study, 35% of the patients enrolled were admitted into the ICU postoperatively. In a 2015 study by Banerjee et al., 70% of the patients undergoing emergency laparotomy required a high-dependency unit bed or critical care [14]. Emergency Laparotomy Collaborative (ELC) and the Emergency Laparotomy Pathway Quality Improvement Care (ELPQuiC) bundle project suggest that all patients should be admitted to the ICU after emergency laparotomy. However, due to the paucity of available ICU beds at our institution, the recommendation could not be followed. Kaafarani et al. in their study demonstrated the applicability of ESS in predicting postoperative ICU admission. Fifty-seven percent of patients required ICU admission postoperatively. ESS accurately predicted which patients required ICU admission (c-statistic, 0.80) with high ESS requiring critical care [13]. Our study also showed a similar trend with c-statistic of 0.87.

ESS and reoperation and readmission

In our study, three (15%) patients underwent reoperation. ESS score showed a poor correlation with the requirement of reoperation within 30 days of emergency laparotomy (c-statistic = 0.667). In a recent study by Kassahun et al., 35.9% of patients required subsequent reoperation after emergency laparotomy, and 547 (64.1%) did not. The incidence of postoperative complications was higher in reoperated patients (100%) than in non-reoperated patients (58.9%). There were 305 deaths, with an overall in-hospital mortality rate of 35.7%; 175 (57%) occurred in the re-operated group, and 130 (23.8%) occurred in the non-re-operated group [15]. However, in our study, no significant correlation was found between reoperation requirement and mortality (p-value = 0.30).

Two (10%) patients had to be readmitted within 30 days of emergency laparotomy in our study. ESS had no significant correlation with readmission rates (c-statistic = 0.55). According to Kongkaew Paisan et al., out of 1,347 patients included, 234 (17.4%) had an unplanned readmission within a 30-day postoperative period of emergency laparotomy [16]. The predictors for unplanned readmission included patient factors (e.g., disseminated cancer [odds ratio: 2.22, P = 0.002], weight loss >10% in the past six months [odds ratio: 1.65, P = 0.023], dyspnea at baseline [odds ratio: 1.62, P = 0.026], wound complications [odds ratio: 2.23, P < .001], and discharge to nursing homes [odds ratio: 1.68, P = 0.044] similar to those included in calculating ESS. Readmission, however, was not a significant predictor of mortality according to our study.

ESS has been validated by several studies and found to be useful in patient counseling, prognostication of family members, and benchmarking the quality of emergency surgical interventions [17].

Limitations

Limitations of our study are small sample size, single-center study, non-randomized, only acute abdomen cases are involved and an indication of reoperation, and readmission were not recorded. White race included as one of the parameters in calculating ESS could not be used in our study. The study was carried out among the Indian population without a correlation of race. Infrastructural limitations in the form of a lack of ICU beds and delayed availability of operation theaters may have a corroborative effect on mortality and morbidity.

## Conclusions

Our study demonstrates that ESS effectively predicts postoperative mortality, morbidity, and ICU duration with statistical significance. Additionally, ESS serves as a reliable indicator for the development of postoperative complications such as LRTI, sepsis, DIC, and AKI. Notably, several complications including LRTI, wound dehiscence, sepsis, AKI, thrombocytopenia, DIC, and prolonged ICU stay are significantly associated with mortality. Therefore, our findings support the implementation of risk stratification using the ESS for patients undergoing emergency laparotomy, enabling proactive measures for resuscitation and patient stabilization preoperatively. Further studies with larger sample sizes are required to establish the effectiveness of the ESS scores and comparing with other risk-scoring tools like P-POSSUM (Portsmouth Physiological and Operative Severity Score for the enumeration of Mortality and Morbidity) and APACHE II (Acute Physiology and Chronic Health Evaluation II).
